# Polysomnographic parameters predicting positive airway pressure adherence

**DOI:** 10.1007/s11325-025-03507-9

**Published:** 2025-11-10

**Authors:** Nattarin Nilrat, Wish Banhiran, Phawin Keskool, Chawanont Pimolsri, Navarat Kasemsuk

**Affiliations:** 1 Department of Otorhinolaryngology, Faculty of Medicine, Siriraj Hospital, Mahidol University, 2 Wanglang Road, Bangkok Noi, Bangkok, 10700 Thailand; 2 Department of Otorhinolaryngology, Faculty of Medicine, Prince of Songkla University, Hat Yai, Thailand; 3 Siriraj Sleep Center, Faculty of Medicine, Siriraj Hospital, Mahidol University, Bangkok, Thailand

**Keywords:** Obstructive sleep apnea, PAP adherence, Polysomnography, REM-related OSA

## Abstract

**Purpose:**

To identify polysomnographic predictors of adherence to positive airway pressure (PAP) therapy in adults with obstructive sleep apnea (OSA).

**Methods:**

We performed a retrospective cohort study of adults with moderate to severe OSA who underwent full-night diagnostic polysomnography and initiated PAP therapy. Participants with previous PAP titration studies were excluded. Baseline demographic and polysomnographic variables were extracted, and adherence was defined as PAP use ≥ 4 h/night on ≥ 70% of nights. Logistic regression identified factors independently associated with adherence.

**Results:**

Among 100 enrolled patients, 61 met the adherence criteria. Compared with adherent patients, nonadherent patients did not differ in apnea–hypopnea index, oxygen desaturation index, lowest oxygen saturation, or low arousal threshold. Rapid eye movement–related OSA was significantly more prevalent in the nonadherent group and was the only independent predictor of nonadherence (adjusted odds ratio, 2.52; 95% confidence interval, 1.04–6.22; *p* = 0.033).

**Conclusions:**

Rapid eye movement–related OSA was the only independent predictor of poor adherence to PAP therapy. Sleep stage–specific phenotyping may therefore assist in personalizing adherence-enhancing strategies. Prospective studies are warranted to validate these findings and evaluate targeted interventions.

## Introduction

Obstructive sleep apnea (OSA) is highly prevalent and is associated with cardiovascular morbidity, mortality, and other systemic complications [1]. Positive airway pressure (PAP) therapy is the first-line intervention and markedly improves OSA outcomes [2]. However, many patients demonstrate suboptimal adherence, attenuating the therapy’s cardiovascular benefit [3]., 

Investigators have explored determinants of PAP adherence to facilitate personalized management. Reported predictors span socioeconomic status, psychological factors, comorbidities, and polysomnographic (PSG) characteristics [4–7]. Among PSG measures, the apnea–hypopnea index (AHI), oxygen desaturation index (ODI), lowest oxygen saturation (LSAT), and the percentage of sleep time with oxygen saturation below 90% (T90) have each been associated with PAP adherence [5, 6, 8, 9]. Nevertheless, results across studies are inconsistent.

Low arousal threshold (low ArTH), a proposed pathophysiological trait within the PALM classification of OSA phenotypes (P: loop gain, A: arousal threshold, L: upper airway collapsibility, M: muscle responsiveness), has recently emerged as a potential negative predictor of adherence [9, 10]. Although no standardized clinical measure of ArTH exists, a simplified estimation based on PSG composite scores has been described; this method still requires several computational steps.

This study, therefore, sought practical, easily determined predictors of adherence, with emphasis on PSG variables that can inform individualized OSA management.

## Materials and methods

### Study design and ethical approval

This single-center retrospective cohort study included patients diagnosed with OSA between April 2022 and April 2023. Eligible individuals had initiated continuous PAP (CPAP) therapy and attended at least 1 follow-up visit at Siriraj Hospital.

The Siriraj Institutional Review Board approved the protocol (reference: Si 396/2023). The investigation complied with the Strengthening the Reporting of Observational Studies in Epidemiology guidelines.

### Study population

Adults aged ≥ 18 years who underwent full-night polysomnography demonstrating an AHI ≥ 15 events/h and who received PAP therapy with at least 1 month of follow-up were included. Patients with previous PAP exposure or those treated with sleep surgery, oral appliance therapy, or other conservative interventions were excluded.

### Polysomnography

Polysomnography was performed according to a standardized protocol. Recording channels included electroencephalogram, electrooculogram, electromyogram, electrocardiogram, abdominal and thoracic respiratory movements, pulse oximetry, nasal airflow measured with a pressure transducer, and an oronasal thermistor.

Certified sleep technicians scored each study, and board-certified sleep specialists reviewed the results according to the American Academy of Sleep Medicine Scoring Manual, version 2.6 (2020 update). Apnea was defined as a ≥ 90% reduction in airflow from baseline for at least 10 s, measured with the oronasal thermistor. Hypopnea was defined as a ≥ 30% reduction in airflow for at least 10 s measured with the nasal pressure transducer and associated with either ≥ 3% oxygen desaturation or an electroencephalogram arousal [11]. 

### Outcome measures

Baseline demographic and clinical variables, including comorbidities, were retrieved for every participant. Daytime sleepiness was quantified with the validated Thai version of the Epworth Sleepiness Scale, which assesses the likelihood of dozing in 8 common daytime situations [12]. CPAP adherence was defined as device usage on ≥ 70% of nights each week, with ≥ 4 h of use per night [13]. Adherence data were collected at the 1-month and 3-month follow-up visits.

PSG variables extracted included sleep efficiency, arousal index, AHI, ODI, LSAT, T90, hypopnea–apnea ratio, positional‑related OSA, and rapid eye movement (REM)–related OSA. Positional‑related OSA was defined as an AHI in the supine position that was at least twice that in the nonsupine position. REM-related OSA was present when overall AHI ≥ 5 events/h and REM-AHI was at least twice the non-REM value [14]. Low ArTH was identified when participants met at least 2 of the following criteria: AHI < 30 events/h, LSAT >82.5%, and hypopnea proportion >58.3% of total respiratory events [9]. 

### Statistical analysis

Normally distributed continuous variables were expressed as mean ± standard deviation, whereas nonnormally distributed data were summarized as median (interquartile range). Categorical variables were reported as counts (n) with percentages. Associations among baseline characteristics, PSG parameters, and CPAP adherence were examined with the χ² test, *t* test, or Fisher’s exact test, as appropriate. Depending on the outcome type, logistic or multiple linear regression analyses were performed. A 2-tailed *p* < 0.05 was considered statistically significant. All analyses were performed with R statistical software, version 4.3.2 (R Foundation for Statistical Computing, Vienna, Austria).

## Results

**Fig. 1:**
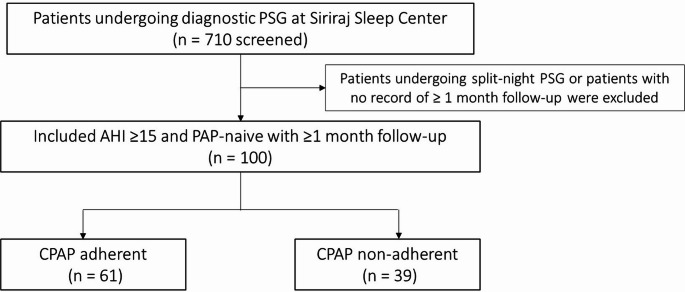
Flowchart of patient selection. Patients who underwent diagnostic polysomnography between April 2022 and April 2023 were screened. After applying inclusion and exclusion criteria, 100 PAP-naive adults with moderate to severe OSA and at least one follow-up visit were included in the final analysis

The cohort comprised 100 participants—56 men and 44 women—with a median age of 61 years (range: 22–87). Sixty-one met the CPAP adherence criterion and formed the adherent group; 39 were nonadherent. Figure 1 illustrates the patient selection process. Baseline characteristics appear in Table 1 and did not differ significantly between the 2 groups. The adherent group recorded marginally higher Epworth Sleepiness Scale scores, but the difference was not significant.

**Table 1:** Baseline characteristics of participants stratified by PAP adherence status (*N* = 100)

	Adherence (*N* = 61)	Nonadherence (*N* = 39)	*p* Value
Age, y†	61 (47–68)	62 (49–68)	0.821
Male, n (%)	33 (54%)	23 (59%)	0.900
BMI, kg/m^2^†	27 (24–30)	28 (26–31)	0.384
Underlying diseases, n (%)			
• Hypertension	22 (36%)	18 (46%)	0.315
• Diabetes mellitus type II	12 (20%)	7 (18%)	0.830
• Dyslipidemia	20 (33%)	7 (18%)	0.103
ESS†	7.0 (3.5–10.0)	7.0 (2.0–12.0)	0.855
**Polysomnographic data**			
Sleep efficiency, %†	78 (66–83)	78 (64–88)	0.980
AHI, events/h†	30 (22–37)	28 (21–36)	0.603
ODI, events/h†	16 (10–25)	15 (10–26)	0.674
LSAT, %†	84 (80–88)	84 (79–88)	0.710
T90, %†	1 (0–4)	2 (0–3)	0.980
HAR†	8 (3–26)	5 (2–15)	0.085
Low ArTH, n (%)	41 (67%)	29 (74%)	0.447
Arousal index, events/h‡	26 ± 9	25 ± 13	0.670
Positional-related, n (%)	17 (28%)	13 (33%)	0.561
REM-related, n (%)	13 (21%)	16 (41%)	0.034
**PAP devices**			
PAP brand			0.237
• Brand I	49 (83%)	27 (73%)	–
• Brand II	10 (17%)	10 (27%)	–
PAP type			1.000
• Auto-titrating	53 (91%)	35 (92%)	–
• Fixed pressure	5 (8.6%)	3 (7.9%)	–

† Values are presented as median (IQR); ‡ values are presented as mean (SD)

Abbreviations: AHI, apnea–hypopnea index; ArTH, arousal threshold; BMI, body mass index; ESS, Epworth Sleepiness Scale; HAR, hypopnea-to-apnea ratio; LSAT, lowest oxygen saturation; ODI, oxygen desaturation index; PAP, positive airway pressure device; T90, percentage of time spent with oxygen saturation below 90%

Among the included patients, 88% were treated with auto-adjusting positive airway pressure (autoPAP), 8% received fixed-pressure CPAP, and 4% had no documented pressure mode. All patients were prescribed a nasal mask as the interface. Standard clinical support was provided during routine follow-up visits, but no additional interventions or adherence-enhancing strategies were implemented. There were no significant differences in adherence rates based on device brand, operating mode, or mask type.

**Fig. 2:**
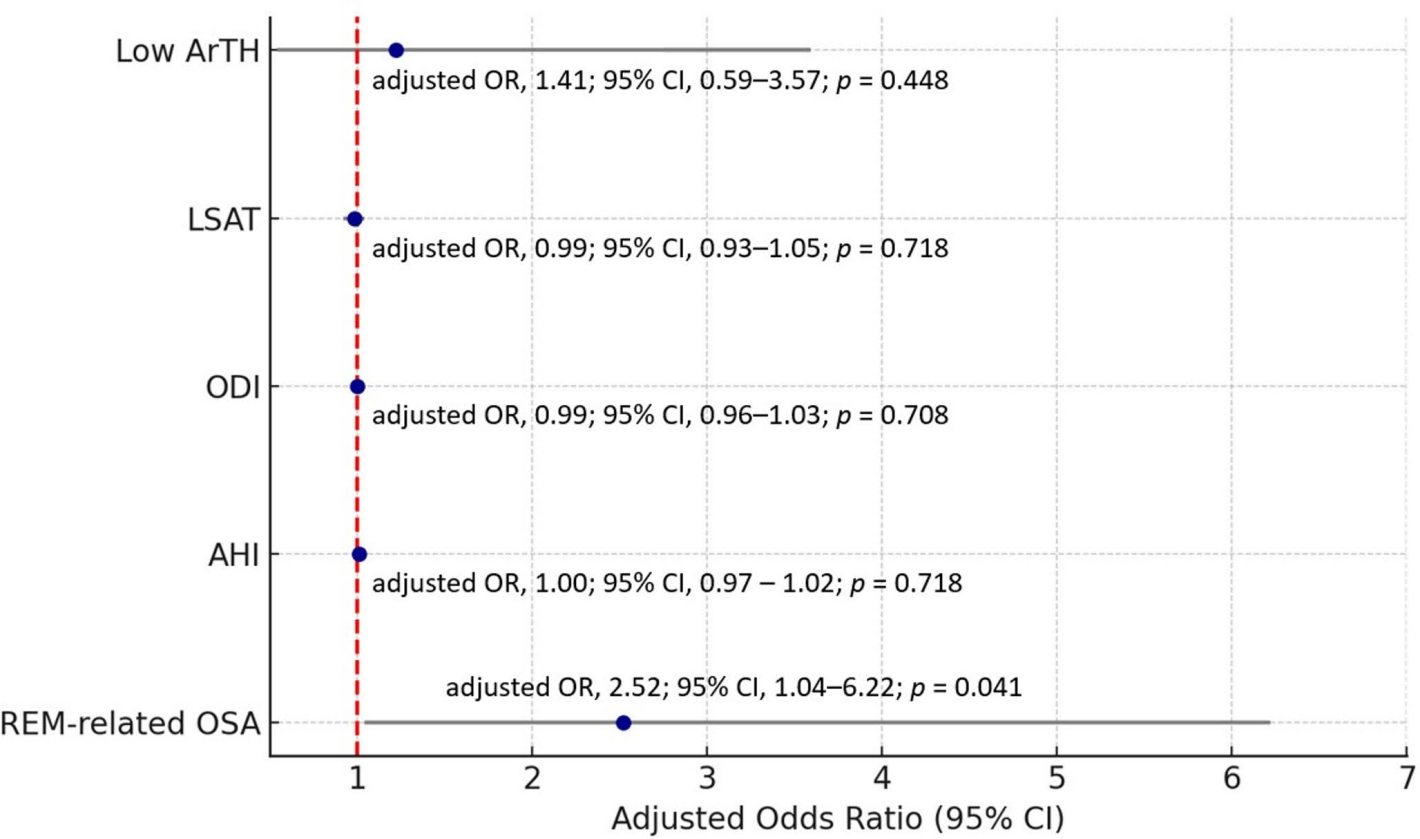
Forest plot of multivariable logistic regression analysis. Adjusted odds ratios(OR) and 95% confidence intervals (CI) are shown for each predictor of PAP adherence. REM-related OSA was significantly associated with nonadherence. Abbreviations: OSA, obstructive sleep apnea; AHI, apnea–hypopnea index; ODI, oxygen desaturation index; LSAT, lowest oxygen saturation; ArTH, arousal threshold

In univariate and multivariate logistic models, REM-related OSA was the only significant predictor of nonadherence (adjusted odds ratio, 2.52, 95% confidence interval, 1.04–6.22; *p* = 0.033). No other PSG variables—including sleep efficiency, arousal index, AHI, ODI, LSAT, T90, hypopnea–apnea ratio, low ArTH, or positional OSA—were associated with adherence (Table 2). Figure 2 presents a forest plot summarizing the results of the multivariable logistic regression analysis, displaying adjusted odds ratios with 95% confidence intervals for each predictor.

**Table 2:** Univariate and multivariate logistic regression of polysomnographic predictors of PAP nonadherence

Univariate analysis	Crude odds ratio	95% CI	*p* Value
Sleep efficiency	0.99	0.96–1.02	0.561
Arousal index	0.99	0.95–1.03	0.645
AHI	1.00	0.97–1.02	0.718
ODI	0.99	0.96–1.03	0.708
LSAT	0.99	0.93–1.05	0.718
T90	0.97	0.92–1.02	0.282
Positional-related	1.29	0.54–3.09	0.561
REM-related	2.57	1.07–6.32	0.037
HAR	0.99	0.97–1.00	0.095
Low ArTH	1.41	0.59–3.57	0.448
**Multivariate analysis**	**Adjusted odds ratio**	**95% CI**	***p*** **Value**
REM-related	2.52	1.04–6.22	0.033
HAR	0.99	0.97–1.00	0.063
Low ArTH	1.33	0.54–3.40	0.405

Abbreviations: AHI, apnea–hypopnea index; ArTH, arousal threshold; CI, confidence interval; HAR, hypopnea-to-apnea ratio; LSAT, lowest oxygen saturation; ODI, oxygen desaturation index; REM, rapid eye movement; T90, percentage of time spent with oxygen saturation below 90%

.

A subgroup analysis contrasted participants with versus without REM-related OSA (Table 3). The REM-related group tended to be younger and included a higher proportion of women, although these differences were not significant. This phenotype exhibited a significantly lower AHI and a lower LSAT value (more severe desaturation). CPAP adherence did not differ by device brand or operating mode.

**Table 3:** Demographic and polysomnographic characteristics of participants with and without REM-related OSA

	No REM-related OSA (*N* = 71)	REM-related OSA (*N* = 29)	*p* Value
Age, y†	62 (50–69)	58 (33–67)	0.072
Male, n (%)	42 (59%)	14 (48%)	0.555
BMI, kg/m^2^†	27 (24–30)	30 (27–34)	0.013
AHI, events/h†	32 (22–42)	23 (21–30)	0.004
ODI, events/h†	17 (9–28)	14 (10–19)	0.401
LSAT†	85 (81–89)	82 (78–86)	0.035
T90†	1 (0–3)	2(0–3)	0.135
Positional-related, n (%)	23 (32%)	7 (24%)	0.414

† Values are presented as median (IQR)

Abbreviations: AHI, apnea–hypopnea index; BMI, body mass index; ESS, Epworth Sleepiness Scale; IQR,interquartile range; LSAT, lowest oxygen saturation; ODI, oxygen desaturation index; OSA, obstructive sleep apnea; REM, rapid eye movement; T90, percentage of time spent with oxygen saturation below 90%

## Discussion

OSA is known to be a heterogeneous condition, with variability in clinical presentation and treatment outcomes. Determinants of PAP adherence are usually grouped into baseline characteristics, motivational factors, and disease severity. Among these, PSG-derived parameters, which are objective markers, have been widely studied as indicators of disease severity. We assessed whether individual PSG parameters predicted PAP adherence in OSA. Our observed PAP adherence rate was 60.6%, consistent with earlier reports ranging from 40% to 85% [15, 16]. Previous investigations have proposed AHI, ODI, LSAT, T90, REM-related OSA, and low ArTH as predictors, yet results remain inconsistent [6, 8, 9, 15, 17–21]. In our cohort, only REM-related OSA predicted nonadherence; no other PSG variable showed a significant association.

Unlike several earlier studies, we observed no significant differences in AHI, ODI, or LSAT between CPAP-adherent and nonadherent groups. The study enrolled only newly diagnosed, PAP-naive patients who had not undergone any titration procedures, including split-night protocols [22]. As a result, individuals with severe OSA—often detected during split-night studies—were likely underrepresented. Consequently, participants exhibited comparatively mild disease, reflected by lower mean AHI and ODI values and higher LSAT values than those reported previously. These factors may have reduced the predictive value of desaturation-related indices in the present analysis. Our findings illustrate how study design and sample composition can modulate the predictive utility of specific PSG metrics, especially in homogeneous or less severe cohorts.

Low ArTH, previously considered a negative predictor of PAP adherence, showed no significant association with nonadherence in this cohort. Our participants predominantly had mild OSA with hypopnea-predominant events, which may have reduced the influence of a low ArTH. Patients with frequent, mild respiratory events were probably underrepresented, limiting detection of low ArTH effects.

REM-related OSA emerged as the sole significant predictor of poor PAP adherence. These patients displayed lower overall AHI, consistent with REM occupying only 20%–25% of total sleep time and even less during fragmented sleep. We speculate that fewer symptoms during non-REM periods reduce motivation to maintain continuous PAP use. REM-related OSA is more common in women, a group previously reported to have lower adherence rates [23], which may partly account for the higher nonadherence seen here. Collectively, the data indicate that sleep-stage–specific phenotypes affect patient comfort and perceived benefit from PAP more than traditional severity metrics such as AHI. In newly diagnosed, PAP-naive patients, early recognition of REM-related OSA could prompt tailored behavioral counselling or intensified follow-up to enhance long-term adherence. Future adherence programs may therefore consider REM-stage–specific strategies, especially for female or nonsleepy patients.

Although the analysis concentrated on PSG-derived variables, we did not evaluate nonphysiological determinants of adherence—such as socioeconomic status, education level, or psychological readiness—and these unmeasured factors may act as potential confounders.

This study has several limitations. First, its retrospective design may have introduced selection bias. Second, the study restrictions probably underrepresented individuals with severe, apnea-predominant OSA by limiting enrolment to PAP-naive patients and excluding those who underwent titration studies. This narrowed the range of PSG metrics and limited detection of associations reported in previous studies. Third, while we emphasized objective PSG data, we could not account for mask comfort or other patient-reported factors, and low ArTH was estimated with a surrogate scoring method rather than direct physiological measurement, potentially affecting its accuracy. Moreover, the study included 100 patients, with only 39 nonadherence events distributed across 10 predictors in the logistic regression model. This yields fewer than 5 events per variable, which can increase the risk of overfitting and reduce the reliability of the odds ratios. The small effective sample size means the result should be interpreted cautiously and validated in larger studies. Finally, the 1- to 3-month observation period was brief and may not have captured the long-term adherence trajectories.

Future investigations should aim to validate these findings in larger, multicenter prospective cohorts with longer follow-up periods. Including psychological profiles, treatment motivation, and patient-reported outcomes could yield a more comprehensive model of adherence behavior. Phenotype-driven strategies, particularly for REM-related OSA or OSA with low ArTH, deserve further study to support tailored interventions and improve outcomes.

## CONCLUSIONS

REM-related OSA was the only PSG parameter significantly associated with nonadherence to PAP therapy. By contrast, widely cited indices—AHI, ODI, LSAT, and low ArTH—were not predictive in this cohort. Early identification of REM-related OSA may prompt clinicians to provide additional support, consider alternative therapies, or tailor adherence interventions accordingly.

## Data Availability

The data supporting the findings of this study are available from the corresponding author on reasonable request but are not publicly shared owing to sensitivity concerns.
